# A Dataset of Microelectrode Recordings from Deep Brain Stimulation Procedures

**DOI:** 10.1038/s41597-026-07492-w

**Published:** 2026-06-10

**Authors:** Katarzyna Osowska, Julian Szymański, Witold Libionka

**Affiliations:** 1 Gdansk University of Technology, Faculty of Electronics, Telecommunications and Informatics, Gdańsk, 80-233 Poland; 2 GUMED, Gdańsk, Poland

**Keywords:** Engineering, Neuroscience

## Abstract

Precise intraoperative localisation of subcortical brain structures remains a critical challenge in deep brain stimulation, yet openly available microelectrode recording datasets are scarce. We present a dataset of 6,646 processed MER recordings from 132 patients with neurological disorders, including Parkinson’s disease, dystonia, Huntington’s disease, epilepsy and others, acquired during DBS procedures. Signals were band-pass filtered and cleaned using an automated machine learning-based artifact rejection pipeline; annotation quality was confirmed by independent review. In addition an experienced electrophysiologist annotated representative examples of three basal ganglia structures encountered along the electrode trajectories: striatum/putamen, external globus pallidus (GPe), and internal globus pallidus (GPi). The dataset, released together with the full processing pipeline and metadata, is intended to support semi-supervised subcortical structure classification, pathological neuronal activity analysis, and the development of novel DBS targeting methods.

## Background & Summary

Deep brain stimulation (DBS) has emerged as an effective therapeutic approach for drug-resistant neurological and psychiatric disorders, with well-documented benefits in the treatment of Parkinson’s disease^1–3^. It is a progressive movement disorder characterized by the degeneration of dopaminergic neurons, leading to motor symptoms such as tremor, rigidity, and bradykinesia. Given the limited efficacy of available pharmacological treatments and the broad therapeutic potential of DBS, its application is increasingly being explored across a range of neurodegenerative and psychiatric conditions^4^.

Despite its clinical success, precise intraoperative localization of the target brain structure remains one of the most critical challenges in DBS. The intended target is typically only a few millimeters in diameter, making accurate electrode placement essential for therapeutic outcomes. During the procedure, a microelectrode is progressively and linearly advanced into the brain, with microelectrode recordings acquired at defined depths relative to a preoperatively established MRI-based target. These electrophysiological signals reflect the characteristic firing patterns of individual neurons and local field potentials, providing real-time feedback on the electrode’s position relative to surrounding subcortical structures. In the recordings presented here, to increase the probability of reaching the target region, up to five parallel microelectrodes arranged in a standard Ben-Gun configuration may be deployed simultaneously, each recording on a separate channel^5^. The same microelectrode assembly can subsequently be used to deliver therapeutic electrical stimulation to the identified target.

Here we present a dataset of MER recordings acquired during DBS procedures targeting the GPi and other subcortical structures in 132 patients with clinically defined diagnoses, including Parkinson’s disease, dystonia, epilepsy, Huntington’s disease, and other neurological dysfunctions. Open-access MER datasets accompanied by expert annotations of traversed brain structures remain scarce, limiting the reproducibility and comparability of research in this field. To address this gap, we release the processed MER alongside a data-processing pipeline designed to support reproducible analyses. The recordings were band-pass filtered to isolate informative frequency components, and artifactual segments were subsequently discarded. Using the Label Studio platform^6^, an experienced electrophysiologist annotated the most representative MER fragments of basal ganglia structures encountered along the electrode trajectories and verified the integrity of the processed signals.

The dataset has been prepared to support a range of future research directions. These include automatic classification of subcortical brain structures for improved intraoperative electrode localization, as well as characterization of pathological neuronal activity patterns across diagnostic groups and patient demographics such as age and sex. Another direction involves the development of novel signal visualization and sonification approaches, exploiting the acoustic-like temporal and spectral properties of MER signals for intraoperative applications.

Given that the vast majority of recordings are unannotated, the dataset is particularly well suited for unsupervised, semi-supervised, and self-supervised representation learning. It can serve, for instance, as pretraining data for foundation models of neural electrophysiology. It is also suitable for training generative models capable of synthesizing novel MER signals and augmenting limited labeled datasets. By releasing both the data and the processing pipeline, we aim to facilitate reproducible, data-driven advances in DBS research and therapy optimization.

## Methods

### Participants and ethics

A total of 132 participants were included in the study (61 women, 46.2%; 71 men, 53.8%). The mean age was 53.5 ± 14.1 years (range: 9–75). All recordings originate from DBS procedures performed at a single clinical center, the University Clinical Centre in Gdańsk, Poland. Clinical diagnoses were established according to the ICD-10 classification. The most prevalent diagnosis was Parkinson’s disease (G20; *n* = 63, 47.7%), with DBS targeting the STN or GPi, followed by Huntington’s disease (G10; *n* = 17, 12.9%) targeting both the GPe and GPi, other extrapyramidal and movement disorders (G25; *n* = 7, 5.3%) targeting VIM and ZI, and dystonia (G24; *n* = 4, 3.0%) targeting GPi. Less frequent diagnoses included epilepsy (G40; *n* = 2, 1.5%) targeting ATN, parkinsonism in diseases classified elsewhere (G22; *n* = 1, 0.8%) targeting STN or GPi, other degenerative diseases of the nervous system in diseases classified elsewhere (G29; *n* = 1, 0.8%), and abnormal involuntary movements (R25; *n* = 1, 0.8%). For n = 36 participants, the clinical diagnosis was not available in the archived documentation at the time of dataset curation. The original MER recordings were preserved, but the associated clinical records could not be reliably retrieved from historical archives. These recordings were retained in the dataset because the signal data themselves remained available and suitable for analyses not requiring diagnosis or target annotations. In the metadata table, the diagnosis and target fields for these participants are marked as unavailable.

The Ethics Commission of Medical Sciences Department of University of Warmia and Mazury has approved the treatment, granting permission under 43/2016 and its extension has been granted on 14.09.2021 from Bioethical commission of Nicolaus Copernicus University in Toruń under decissions KB 416/2008 and KB 28/2010. All patients signed the Consent Form to take part in the experiment and granted permission for the publication and processing for research purposes recordings taken during DBS treatments.

### MER acquisition and preprocessing

MER recordings were acquired using the ISIS MER system (Inomed Medizintechnik GmbH) with MicroMacro electrodes arranged in a standard Ben-Gun configuration. Signals were amplified with a gain of approximately 10,000× and recorded with hardware band-pass filtering typical for intraoperative MER acquisition. A reference electrode was placed on the scalp, while the ground electrode was positioned on the patient’s chest.

DBS procedures were performed under local anesthesia with the patient awake, allowing intraoperative electrophysiological monitoring. Stereotactic targeting was performed using preoperative MRI and stereotactic planning software. Preoperative targeting was based on T1- and T2-weighted MRI images.

The system includes a MicroDrive enabling insertion of up to five electrodes in a Ben-Gun configuration. MicroMacro electrodes, which allow simultaneous stimulation and recording of neuronal activity, were used. The device stored the acquired signals without on-device preprocessing. Signals from implanted microelectrodes were automatically saved as binary .dat files. Each file corresponds to a single recording obtained at a defined depth for one of the implanted electrodes. The .dat files were accompanied by procedural protocols describing recording settings, including a sampling rate of 20 kHz and 16-bit signed-integer quantization.

**Fig. 1:**
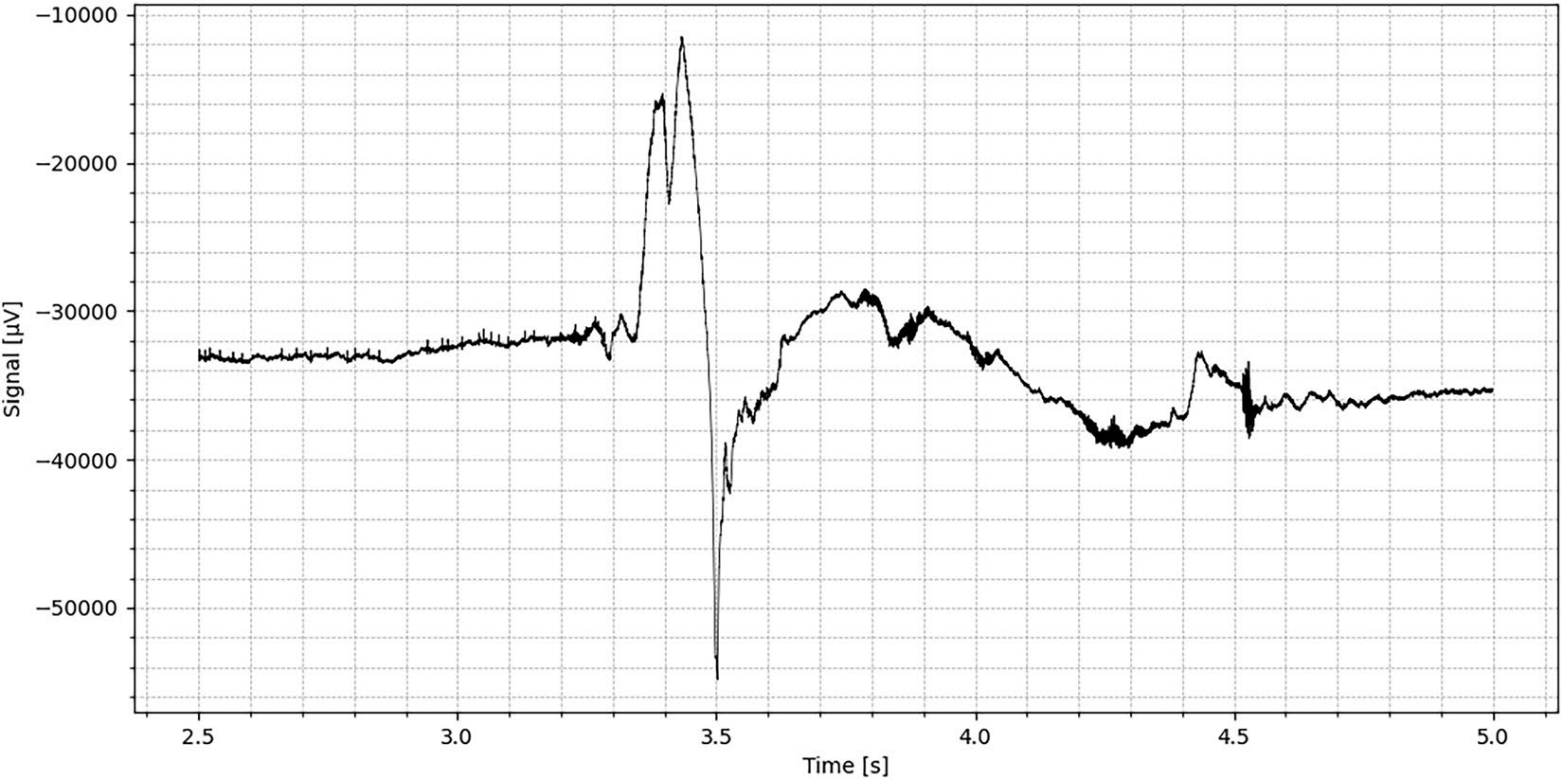
Raw MER.

The MER signals were extracted directly from the original binary files. After skipping a fixed file header (offset 0 × 25C), the data stream was decoded as int16. To prevent numerical overflow in subsequent processing, the samples were converted to float32 and exported to CSV files with two columns: time (s) and amplitude (instrument units). An example visualization of the raw MER is provided in Fig. 1.

We did not have access to manufacturer documentation required for an exact conversion from stored int16 values to physical units. Therefore, amplitudes should be interpreted as instrument units “*μ*V-equivalent”, potentially subject to an unknown global linear scaling factor. Nevertheless, the observed amplitude ranges are consistent with typical intraoperative MER magnitudes: baseline activity is on the order of ~50−100 (instrument units), while spike amplitudes commonly fall within ~100–500, which is compatible with a microvolt-scale interpretation.

**Fig. 2:**
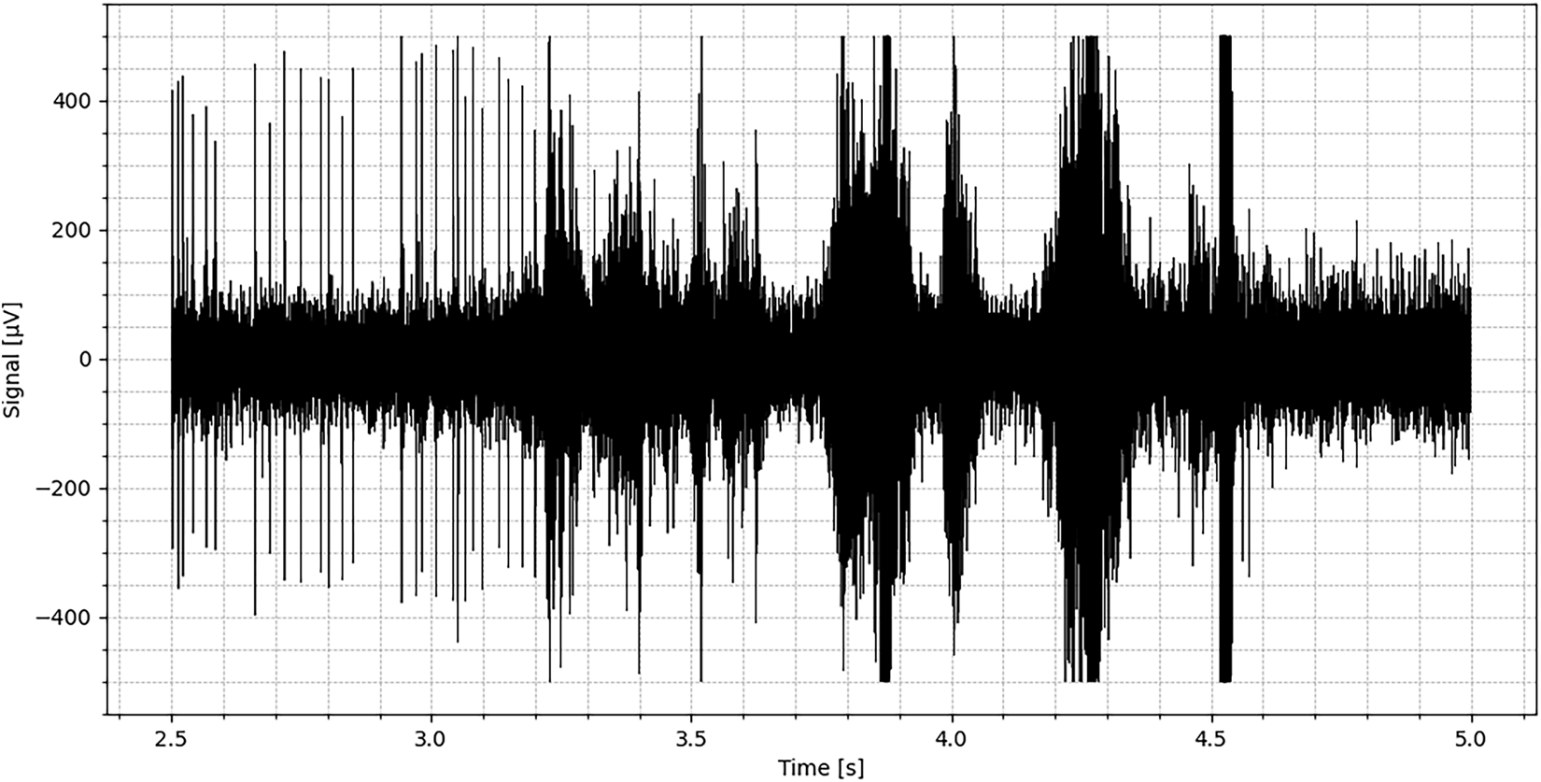
Corresponding to raw MER in Fig. 1 spike-band filtered MER.

Raw recordings contained low-frequency drift, baseline fluctuations, and transient artifacts therefore, preprocessing was applied prior to annotation. signals were band-pass filtered using a fourth-order butterworth filter in the spike band (500–5000 hz). to mitigate the influence of high-amplitude transients associated with movement or electrical interference, signal amplitudes were clipped to ±500 instrument units - a threshold exceeding typical physiological MER ranges and thus indicative of artifactual origin. a comparison of the raw and processed mer signal is shown in Fig. 2. The processed recordings retain the original sampling rate of 20 khz and are stored in float32 precision.

### Subcortical brain structures annotations

**Fig. 3:**
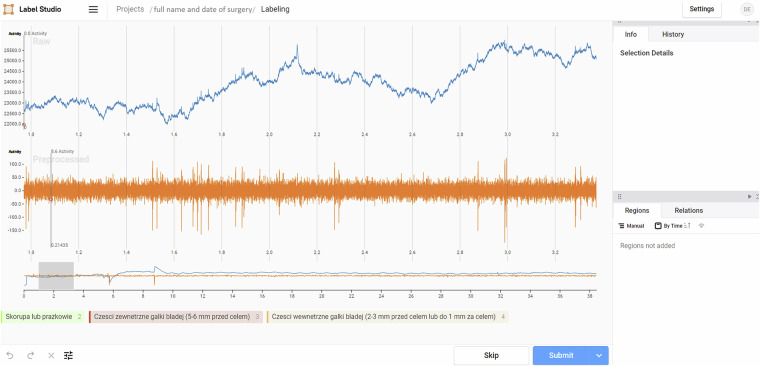
Label Studio interface used for annotation and review of MER signals.

Anatomical structures encountered along each microelectrode trajectory were annotated by an experienced electrophysiologist using the Label Studio platform^6^ (Fig. 3), based on visual inspection of the electrophysiological signals recorded during GPi-targeted DBS procedures. Each recording file corresponds to a single electrode channel acquired at a predefined depth (in mm) relative to the reference target specified in the procedural protocol. Rather than delineating depth-based boundaries between structures, which are inherently diffuse and difficult to assign unambiguously, the annotator labeled continuous time intervals within each recording, selecting segments with clearly visible neuronal activity judged to be representative of the traversed region. Annotation decisions were guided by changes in spike density and background activity, independently of preoperative targeting information beyond the recorded depth. Both the raw wideband signal and the processed spike-band signal were inspected in parallel during the annotation process. Three anatomical classes were annotated along the GPi trajectory: (i) striatum (STR, used interchangeably with putamen, as these structures were not distinguished in the annotations), typically encountered first along the trajectory; (ii) external globus pallidus (GPe); and (iii) internal globus pallidus (GPi), which constituted the surgical target.

Annotated segments are provided in the shared repository under the “brain_layer_annotations” directory as CSV excerpts extracted from the main dataset. Files preserve the same two-column structure (time in seconds and amplitude in instrument units) as the corresponding source recordings and are organized into subfolders named by anatomical class. Table 1 summarizes the number of annotated segments per class. The electrophysiological characteristics of each annotated structure, as identified during the annotation process, are summarized in Table 2 and may serve as a reference for future classification experiments.

**Table 1:** Summary of annotated subcortical brain structures along microelectrode trajectories in GPi-DBS.

	Putamen or Striatum	External Globus Pallidus	Internal Globus Pallidus
Number of annotations	7	10	12
Total duration [s]	8.08	12.45	11.01

**Table 2:** Characteristics of subcortical brain structures in GPi-DBS.

Feature	Striatum or Putamen	External Globus Pallidus	Internal Globus Pallidus
Spike Frequency	low	high	very high
Baseline Relative to Spikes	wide	wide	narrow
Spike Regularity (Achieved Amplitude)	low	medium	high
Tissue Diameter	—	3–4 millimeters	3 millimeters
Distance from Target	far	close (5–6 millimeters before the target)	target
Spike Amplitude	low	high 100–250 [*μ*V]	very high 150–500 [*μ*V]

### Signal quality classification

Although band-pass filtering removed low-frequency drift and noise, the processed recordings still contained transient artifacts that could compromise downstream analyses. To address this, all recordings were segmented and each segment assigned to one of three signal-quality classes.

Recordings typically contained segments of varying signal quality. “Artifact” segments result from mechanical disturbances, for example when the electrode enters the cerebrospinal fluid or shifts relative to the brain. These segments show large, abrupt amplitude changes, such as a sudden increase during electrode displacement or a substantial decrease when in contact with cerebrospinal fluid, and were excluded from subsequent analyses. “Background activity” represents the signal in the absence of detectable neuronal firing, characterized by a stable baseline and a low or zero spike count, and is present throughout most recordings. “Neuronal activity” denotes segments containing prominent spiking activity originating from the structure where the electrode is located, characterized by a high spike count with relatively high amplitude. These segments constitute the primary target for further analysis. Training examples for all three classes were manually annotated in Label Studio and are summarized in Table 3. The annotated segments used for classifier training are provided in the accompanying codes directory, under “signal_quality_classifier_dataset”. Classifier performance is reported in the Technical Validation section.

**Table 3:** Distribution of annotated signal-quality segments used to train the automatic cleaning model.

Measure	Artifact	Neuronal activity	Background signal
Number of annotations	157	63	35
Total duration [s]	41.7	151.3	272.8
Average duration [s]	0.3	2.4	7.8

We developed a signal-cleaning pipeline based on a Random Forest classifier with hyperparameters reported in Table 4. For each 0.5 s non-overlapping segment, we computed a set of time-domain and frequency-domain features capturing spiking activity, spectral content, and distributional shape, following established approaches for automatic artifact detection in MER signals^7^. Spike-related features were derived from detected spikes, defined as local maxima exceeding a threshold of three standard deviations of the segment amplitude, while additional features quantified spectral characteristics and higher-order statistics. Exact feature definitions and implementation details are provided in the accompanying code.

**Table 4:** Random forest hyperparameters and their descriptions.

Parameter	Value	Description
n_estimators	100	Number of trees
criterion	gini	Node impurity measure
max_depth	5	Maximum depth of the tree
min_samples_split	5	Minimum number of samples required to split a node
max_features	sqrt	Number of features to consider when splitting
test size	50%	Proportion of the data used for testing

The pretrained classifier was subsequently applied to all recordings to perform automated cleaning. From each recording, we retained the longest continuous fragment satisfying one of two criteria: at least three consecutive “Neuronal activity” segments (minimum 1.5 s), or the longest artifact-free fragment of at least 2 s. This hierarchical selection prioritizes high-quality neuronal data while ensuring a minimum duration suitable for downstream analyses. Processing statistics are reported in the Technical Validation section.

## Data Records

**Fig. 4:**
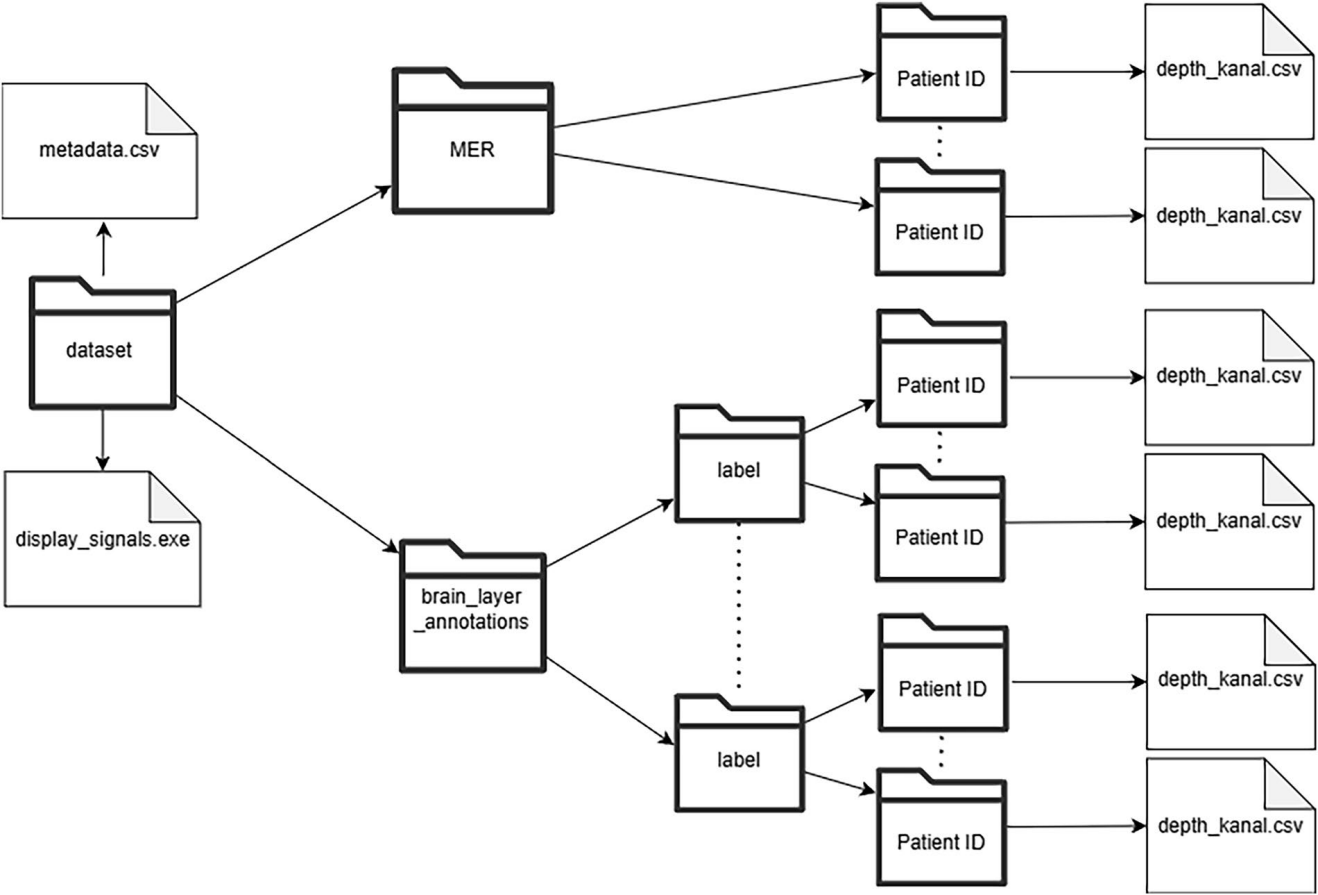
Organisation of the released dataset archive.

The dataset has been deposited in the open repository MOST Wiedzy^8^ and is distributed as a single archive. The archive contains one top-level folder with two ZIP archives: “codes” (preprocessing, machine learning analysis and visualization scripts) and “dataset” summarised in Table 5. The overall organisation of the archive is shown in Fig. 4.

**Table 5:** Overview of the microelectrode recording dataset.

Measure	Value
Number of patients	132
Number of MER recordings	6,646
Number of electrode channels	1–5 per patient
Total recording duration	22.43 h
Mean recording duration	12.15 ± 9.89 s
Median recording duration	10.00 s
Recording duration range	1.54–143.41 s
Sampling rate	20 kHz
Quantization	float32
Total dataset size	44.3 GB

### MER recordings

Within “dataset”, the directory “MER” contains all microelectrode recordings acquired during DBS procedures. Recordings are grouped by participant: each subdirectory in “MER” is named using the patient identifier and contains the corresponding MER traces stored as CSV files. The signals in “MER” correspond to recordings after the full preprocessing pipeline described in Methods. Individual recordings typically span several to tens of seconds and retain the original sampling rate of 20 kHz.

Each MER trace is stored as comma-separated values file (CSV) with two columns: “Time” (in seconds) and “2: preprocessed” (signal amplitude in *μ*V-equivalent units).

Files corresponding to a given electrode trajectory and recording depth are named according to the convention:


depth<distance_from_reference_target_in_millimeters>_kanal<channel_name>.csv


where “distance_from_reference_target_in_millimeters” denotes the depth relative to the reference target (typically starting at − 10 mm; 0.0 mm corresponds to the target, positive values indicate advancement beyond the target), and “channel_name” identifies the microelectrode channel (“central”, “anterior”, “posterior”, “medial”, or “lateral”).

### Brain structure annotations

The “brain_layer_annotations” directory contains CSV files with MER signal segments annotated with brain-structure labels summarized in Table 1. Files are organised hierarchically by anatomical class and patient ID: each top-level subdirectory corresponds to an annotated structure, and contains patient-specific subfolders with CSV excerpts following the same two-column format and naming convention as the source recordings in the MER directory.

### Metadata

Participant-level information is provided in “metadata.csv”. The table includes the following fields: ID, DATE, DIAGNOSIS, GENDER, AGE and TARGET. The ID corresponds to the name of the participant subdirectory in MER. DATE indicates the procedure date (year and month). AGE is the patient age at the time of the procedure. DIAGNOSIS is encoded according to the International Statistical Classification of Diseases and Related Health Problems, 10th Revision (ICD-10). TARGET refers to the brain structure that was the subject of the surgical procedure (e.g., GPi or ATN). This value remains consistent across all patients sharing the same diagnosis. In some cases, the target field contains two values separated by “_AND_”, indicating that the recording spanned two anatomical layers simultaneously with the upper part of the microelectrode positioned in one layer and the lower part in the other. For cases G20 and G22, the target is labeled as STN_OR_GPi in the metadata, indicating that the recordings originated from either STN or GPi, approximately 90% of these recordings are estimated to be STN and 10% GPi.

### Protocols

For each DBS procedure (one per patient), a single acquisition and stimulation protocol was generated and included in the archived record. The protocol file is stored alongside the recordings in the corresponding patient subdirectory within “MER” directory. These protocol files document key recording parameters (sampling frequency, quantisation, and the set of active channels following the Ben-Gun alignment) as well as stimulation settings (current amplitude, frequency, and pulse width). All parameters, including recording depth, are recorded automatically by the acquisition system. Stimulation depth entries constitute an exception: these were transcribed manually during surgery and should be treated as potentially uncertain.

A representative excerpt from a protocol file is shown below:

09:28:23 -- New site no 21 created (Depth: -4,0 mm)

09:28:23 --

09:28:23 -- Quantisation: 16 Bit

09:28:23 -- Sampling rate: 20000Hz, channel configuration: 1: Central, 2: Lateral

09:32:00 -- Stimulation-> Electrode:Ma Channel:1 Depth:-5,2 Current(mA):3,00

Freq(Hz):130 Pulse(us):60

This excerpt encodes (i) recording-site depth relative to the reference target (Depth: -4.0 mm), (ii) acquisition settings (Quantisation: 16 Bit; Sampling rate: 20000Hz; active channels Central and Lateral), and (iii) stimulation settings (channel Central, stimulation depth -5,2, current 3.00 mA, frequency 130 Hz, pulse width 60 *μ*s). In this excerpt, the stimulation depth entry (Depth:-5,2 in the stimulation line) was transcribed manually during surgery and should therefore be treated as potentially uncertain; the recording-site depth (Depth: -4,0 mm) and all remaining acquisition parameters were recorded automatically by the acquisition system.

### MER visualisation software

**Fig. 5:**
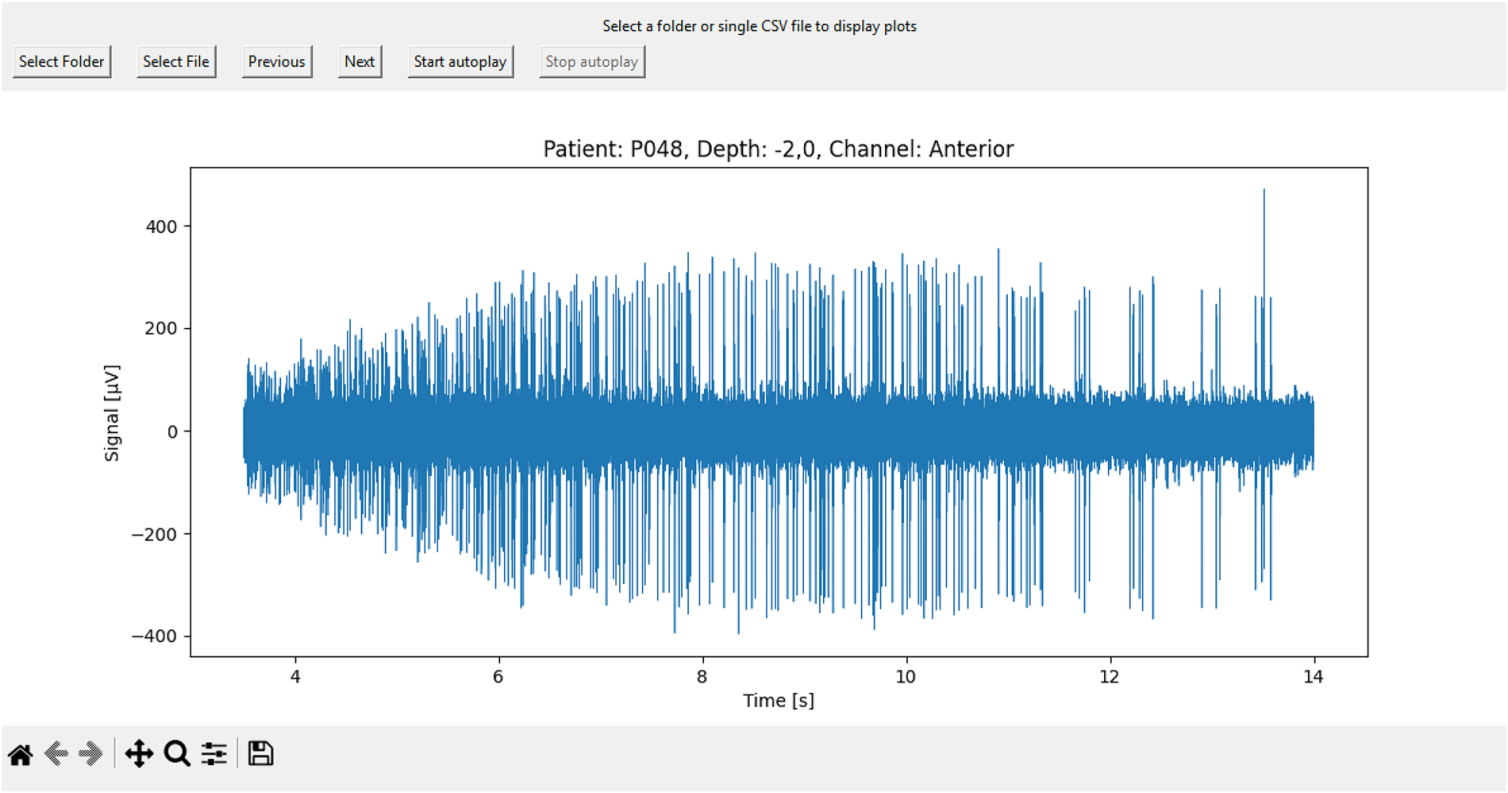
Interface of the included MER visualisation tool.

The archive includes a lightweight program for visualising MER signals (see Fig. 5). The software scans the dataset directories for CSV files containing the “Time” and “2: preprocessed” columns and displays the selected trace as signal amplitude versus time. For each file, the program parses the filename to extract the recording depth and channel label, which are shown in the plot header.

Two browsing modes are provided: (i) an automated mode that advances through consecutive files at 2 s intervals and (ii) a manual mode for file-by-file navigation. The interface supports zooming and panning, basic display customisation, loading a single file, and exporting the current view as a PNG image.

The viewer is intended for rapid data inspection without requiring a Python environment and does not modify the underlying data files.

## Technical Validation

### Preprocessing validation

**Fig. 6:**
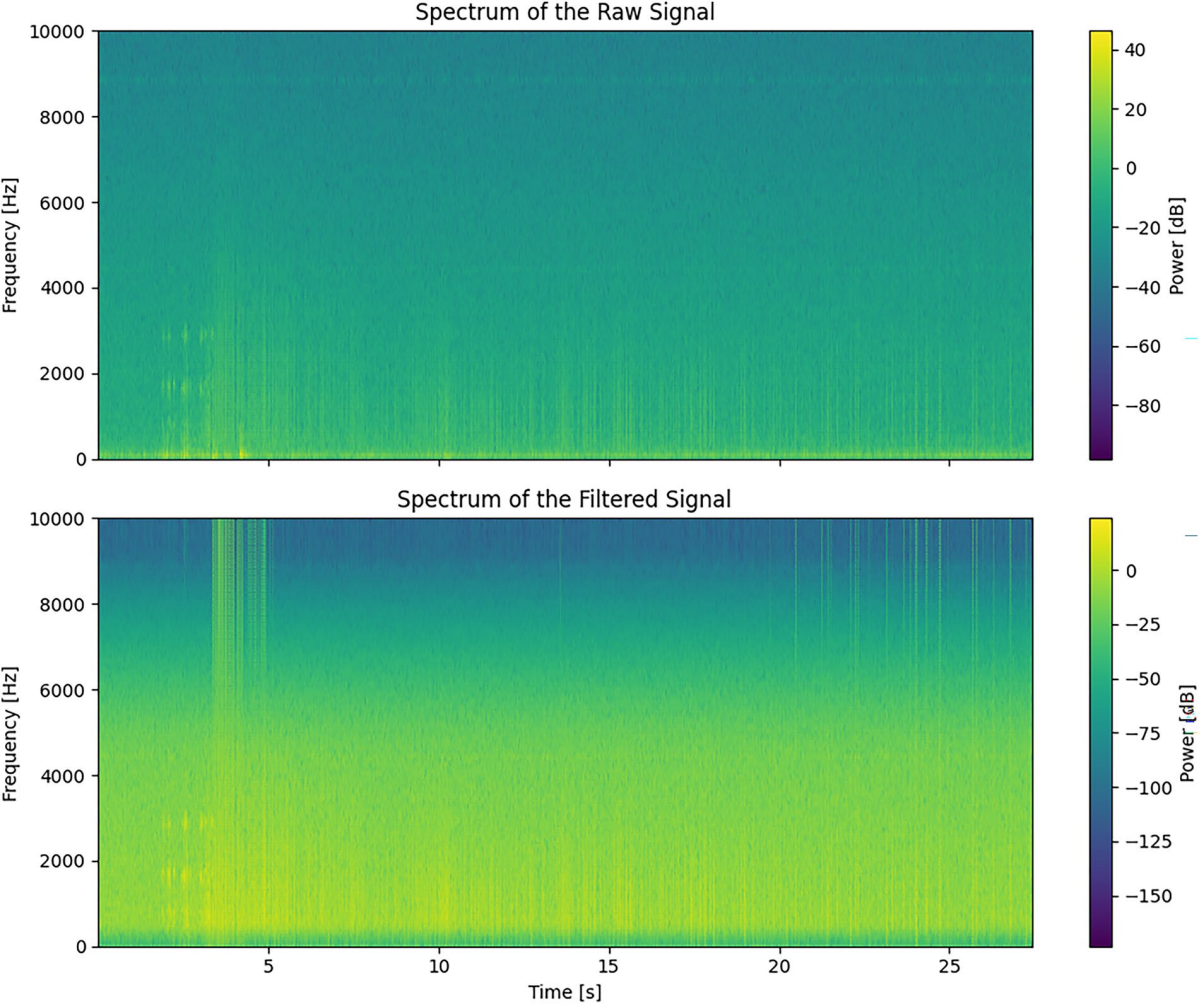
Time–frequency spectrograms of an example MER trace before and after spike-band filtering.

The band-pass filter was designed to replicate the filtered traces displayed by the clinical acquisition system, which saved raw signals while presenting filtered data during clinical use. Raw and filtered traces were inspected side-by-side in Label Studio to facilitate qualitative assessment (Fig. 3). A neurophysiologist confirmed that the processed signals closely resembled those displayed by the acquisition system and remained interpretable for identifying structures of origin. This assessment was further corroborated by comparison of time-frequency representations (spectrograms) of the raw and filtered signals (Fig. 6).

### Artifact removal and quality control

**Fig. 7:**
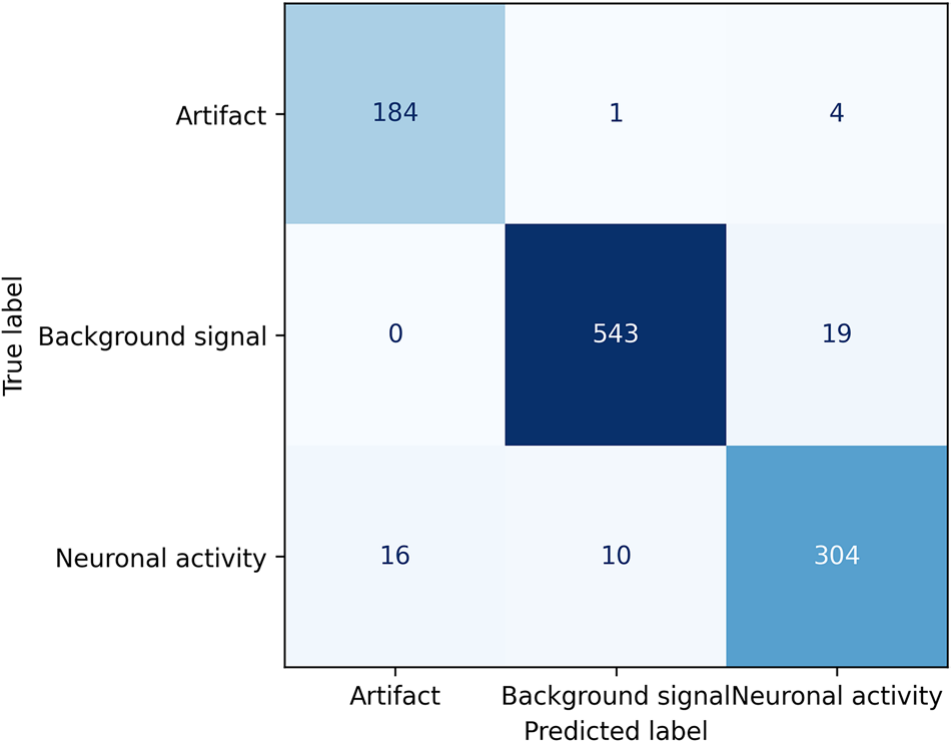
Confusion matrix of the Random Forest artifact classifier evaluated on the hold-out test set.

**Fig. 8:**
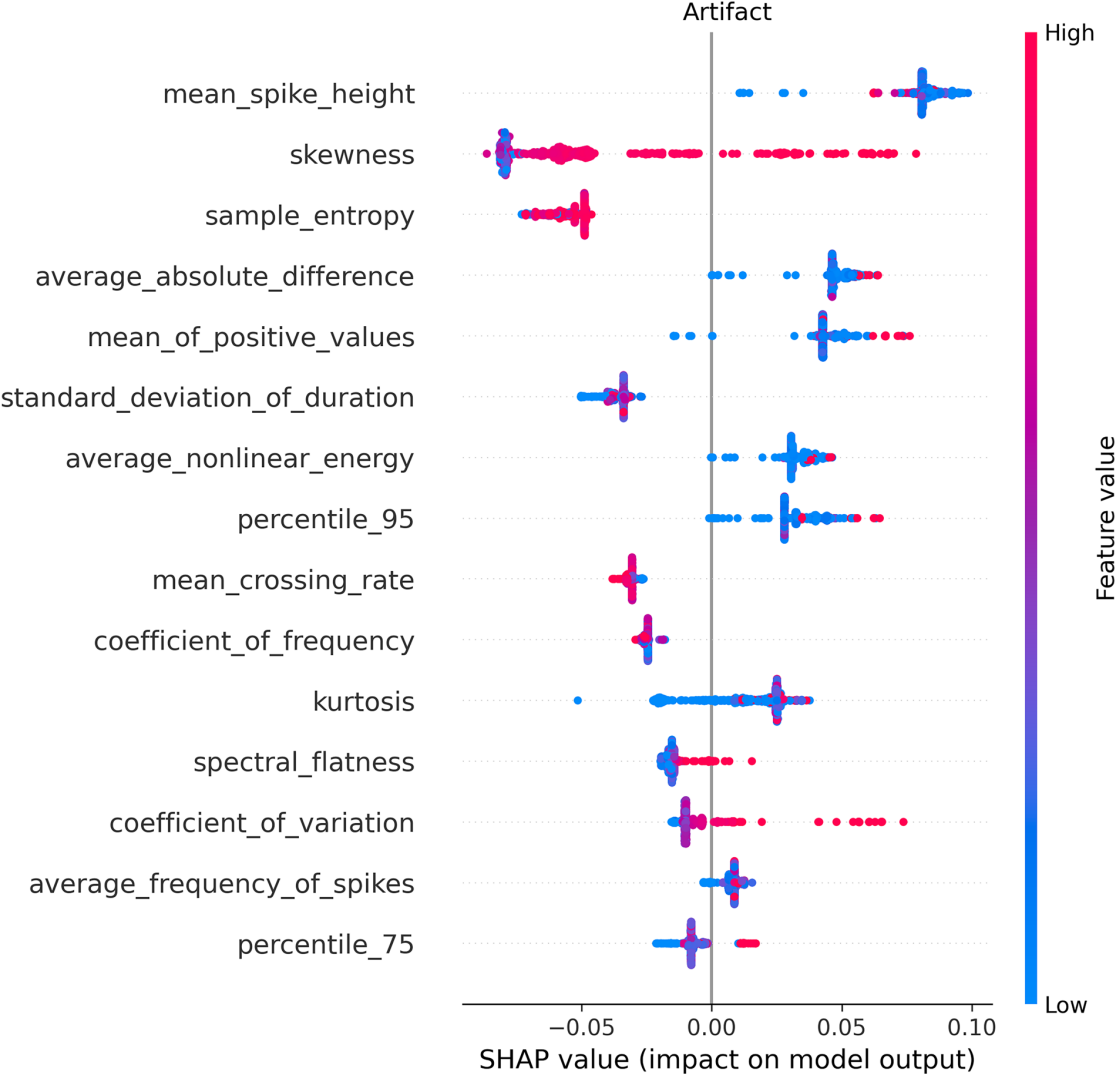
SHAP values for each feature across all Artifact examples displayed as a beeswarm plot.

**Fig. 9:**
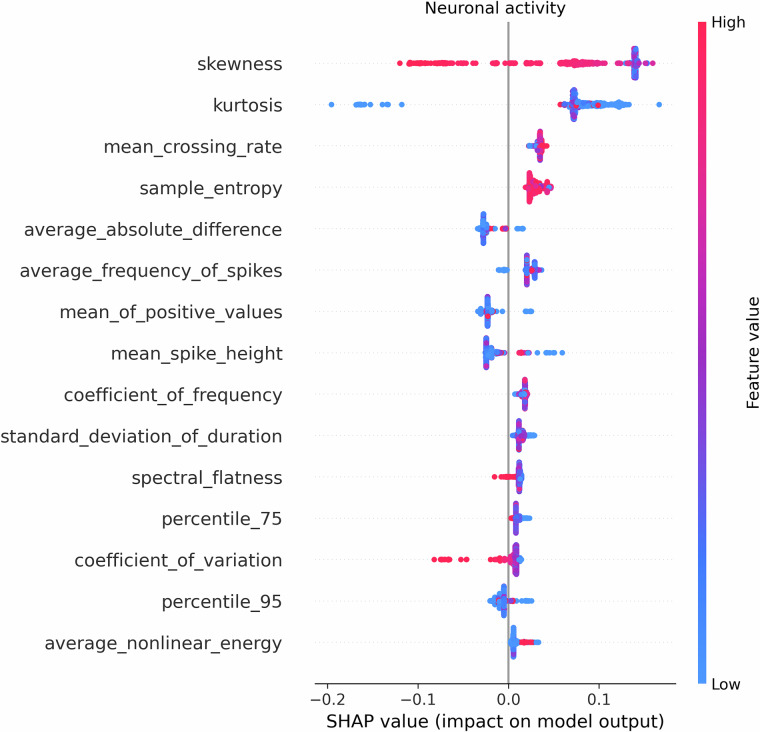
SHAP values for each feature across all Neuronal activity examples displayed as a beeswarm plot.

**Fig. 10:**
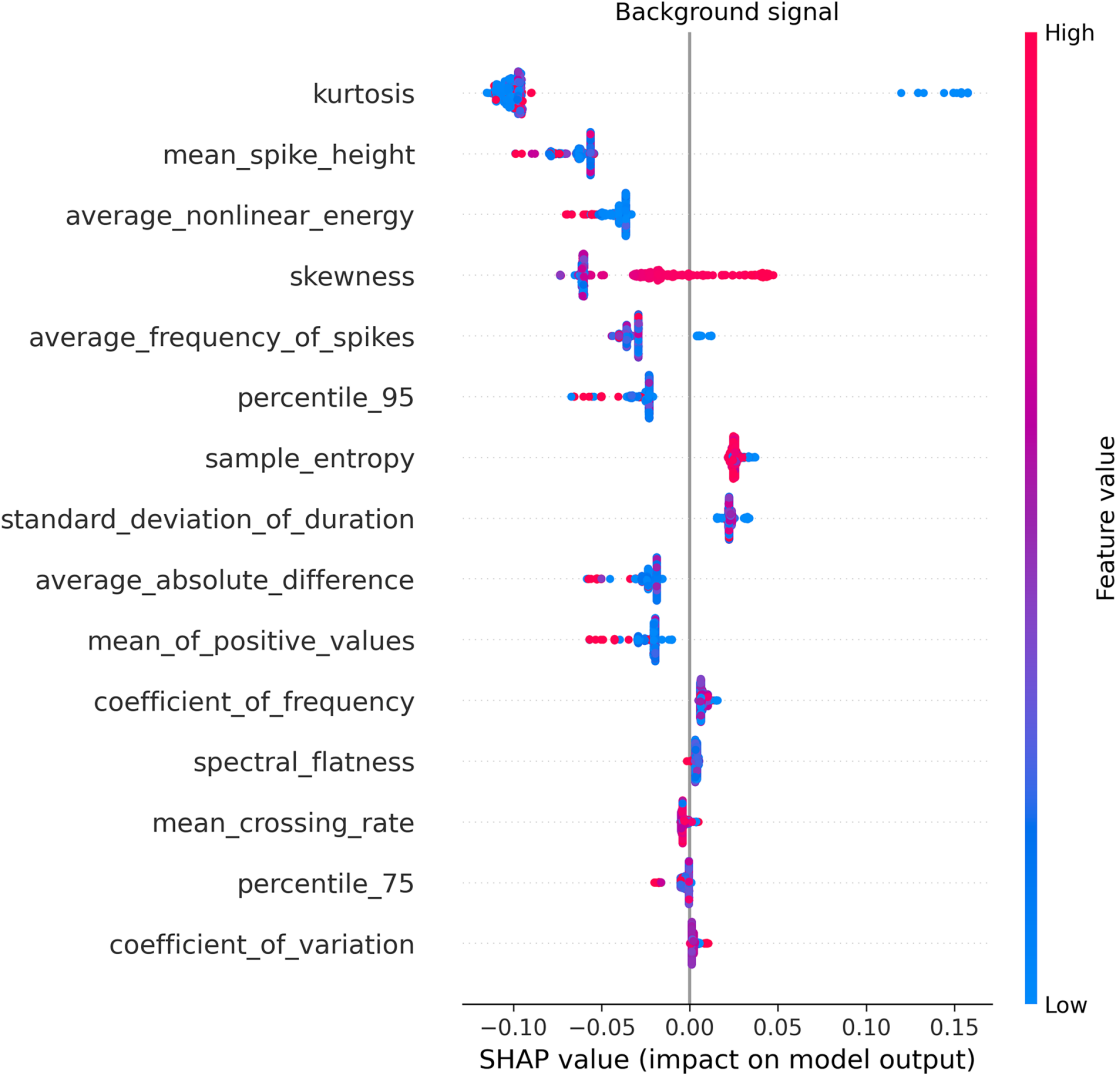
SHAP values for each feature across all Background signal examples displayed as a beeswarm plot.

Feature selection was informed by the domain expertise of the neurophysiologist, who identified signal characteristics relevant to structure classification and artifact recognition. These insights guided the design of engineered temporal and spectral statistics (e.g., kurtosis, skewness, sample entropy, and frequency/energy descriptors) used by an automatic cleaning pipeline based on a Random Forest classifier. It was evaluated using recording-grouped splits, ensuring that all segments originating from the same recording were assigned to a single fold. On a 50% hold-out test set, the model achieved an accuracy of 0.96, a balanced accuracy of 0.95, and a weighted F1 score of 0.96. Performance was highest for Background signal (F1 = 0.99), with slightly lower but consistent performance for Artifact (F1 = 0.93) and Neuronal activity (F1 = 0.93). Most misclassifications occurred between Neuronal activity and Artifact segments, likely reflecting the shared presence of high-amplitude transients in both classes. The confusion matrix based on out-of-fold predictions from the cross-validation is shown in Fig. 7. SHAP values were computed exclusively on the training set to prevent data leakage, and are shown in Figs. 8, 9, 10. In addition, the model was validated using 5-fold group cross-validation (grouped by recording), obtaining a mean balanced accuracy of 0.95 ± 0.02 and a mean weighted F1 score of 0.95 ± 0.02; both evaluations are reported for completeness, with hold-out metrics serving as the primary performance estimate and cross-validation confirming result stability.

After automatic cleaning and trimming, an additional manual spot review was performed to confirm the quality of the final dataset. The most critical failure mode is misclassification of artifact segments as Neuronal Activity or Background Activity, as this would introduce noise into the dataset. Manual inspection indicated that any residual artifacts typically occur as very short fragments (approximately 10 ms) and, relative to the multi-second segments used for analysis, do not constitute a meaningful limitation for expert review or automated processing. To further reduce false positives for neuronal activity, classifications were required to be consistent across three consecutive segments, and a minimum segment duration of 2 s was enforced. Overall, the cleaning process reduced the dataset from 6832 to 6646 files; the corresponding reduction in size from 82.6 GB to 44.2 GB reflects primarily the trimming of low-quality segments rather than the exclusion of entire recordings.

### Validation of subcortical brain structure annotations

The annotator was directly involved in performing the DBS procedures from which the recordings were collected, providing a high level of clinical and anatomical expertise. To ensure annotation quality, two additional reviewers independently inspected all labeled segments; one annotation was identified as incorrect and subsequently excluded. The manually annotated segments are provided primarily as reference examples rather than a comprehensive labeling of the entire dataset. Manual annotation of MER signals requires expert electrophysiological interpretation and is therefore time-consuming. The annotated segments were selected to represent clear and characteristic examples of neuronal activity associated with each structure. These reference annotations may serve as ground-truth examples for algorithm development, feature validation, or supervised learning approaches. Researchers requiring larger labeled datasets may use these segments as seed examples for semi-supervised or weakly supervised labeling strategies.

To verify that the processed signals preserve discriminative characteristics sufficient for structure identification, we trained simple machine learning classifiers to predict the annotated brain structure from signal segments. Correct classification by an independent model provides indirect evidence that the annotations are internally consistent, discrepant cases were reviewed to distinguish labeling errors from model limitations. Due to the limited number of patients per diagnostic group, a cross-patient split was not feasible, instead, a cross-recording split was applied (70% training, 30% test). Segments were split at the recording level prior to windowing, ensuring that overlapping windows derived from the same recording appeared exclusively in either the training or the test set. The signals were segmented into 0.5 s windows with 0.2 s overlap and used to train a Support Vector Classifier (RBF kernel). The model achieved a weighted F1 score of 81.4% and an accuracy of 83.3%, indicating that the recordings contain sufficient information to differentiate the annotated structures. As an alternative baseline, manually engineered temporal and spectral features (e.g., spike rate, spectral flatness, and percentile-based descriptors) were classified using logistic regression, achieving a weighted F1 score of 80.1% and an accuracy of 80.0%. The close agreement between both models confirms that the results are robust and not dependent on the choice of classifier. Detailed implementations are provided in the accompanying code in the “brain_layer_classifier” module (see Section Code availability).

## Usage Notes

The recordings are provided as CSV files and can be analysed using standard Python libraries widely used in scientific computing. We recommend Python (version ≥3.11) with pandas and numpy for data handling and matplotlib for visualisation. An archive includes standalone viewer (“display_signals.exe”) for quick inspection of individual MER traces without requiring a Python environment.

CSV files can be loaded directly into a pandas.DataFrame; the preprocessed signal is stored in the “2: preprocessed” column (see Data Records for file structure details). Basic signal descriptors can be computed with numpy:

import pandas as pd

import numpy as np

csv_data = pd.read_csv(file_path)

signal = csv_data["2: preprocessed"].to_numpy()

std_value = np.std(signal)

A complete worked example, including recursive dataset traversal, signal visualisation, and feature extraction, is provided in the accompanying code repository (see Code availability).

## Data Availability

The complete dataset, including microelectrode recordings, brain structure annotations, patient metadata, and codes is publicly available via MOST Wiedzy^8^ at 10.34808/nq8v-t162.

## Code availability

The source code used for data processing and analysis has been publicly released via MOST Wiedzy^8^. (within the ZIP archive under the codes directory) and on GitHub: https://github.com/kasiaOsowska/Deep-Brain-Stimulation-Microelectrode-Recordings.git. The project requires Python 3.11 with dependencies specified in requirements.txt. The repository includes scripts to (i) convert raw recordings to the distributed CSV format and apply preprocessing, (ii) compute time- and frequency-domain signal metrics and perform automated quality assessment, (iii) prepare MER segments and associated labels for machine-learning workflows (including brain-layer classification), and (iv) support visual inspection and export of example figures.
